# Hepatic Teratoma: An Unusual Presentation of a Germ Cell Neoplasm

**DOI:** 10.7759/cureus.99796

**Published:** 2025-12-21

**Authors:** Muthu M Manickam, Ashwin Kumar, G Murugan

**Affiliations:** 1 Radiodiagnosis, Sree Balaji Medical College and Hospital, Chennai, IND; 2 Radiodiagnosis, Barnard Institute of Radiology, Madras Medical College, Chennai, IND; 3 Radiology, Sree Balaji Medical College and Hospital, Chennai, IND

**Keywords:** case report, ct abdomen, germ cell tumor, liver neoplasm, primary hepatic teratoma

## Abstract

Primary hepatic teratoma is an exceptionally rare germ cell tumor. Its pathogenesis in the liver, an uncommon site for germ cell tumors, remains poorly understood. We present a case of a primary hepatic teratoma in a young adult female to highlight its clinical presentation, radiological hallmarks, and management. A 20-year-old female presented with a five-month history of dull, aching pain in the upper abdomen. Physical examination revealed a non-tender, palpable mass in the right hypochondrium. Routine blood investigations, including liver function tests and serum alpha-fetoprotein, were within normal limits. Contrast-enhanced computed tomography of the abdomen revealed a large, well-defined heterodense mass (~10.2 x 5.3 cm) in the liver. The mass exhibited characteristic heterogeneity with components of fat, calcification, and soft tissue, creating a mass effect on adjacent abdominal vessels. Based on the imaging findings, a provisional diagnosis of a complex benign tumor, likely a teratoma, was made. The patient underwent a successful surgical resection. Histopathological examination confirmed the presence of mature tissues derived from all three germ layers, leading to the definitive diagnosis of a mature cystic teratoma of the liver. This case underscores that primary hepatic teratoma, while exceedingly rare, should be considered in the differential diagnosis of a complex, heterogeneous liver mass, particularly in young patients. Cross-sectional imaging is pivotal in suggesting the diagnosis by identifying pathognomonic elements like fat and calcification. Complete surgical excision remains the cornerstone of treatment and is typically curative for mature lesions, emphasizing the importance of accurate radiological and histopathological correlation.

## Introduction

Teratomas are germ cell tumors characterized by the aberrant proliferation of pluripotent cells, resulting in tissues derived from two or more of the three embryonic germ layers: ectoderm (e.g., skin and neural tissue), mesoderm (e.g., muscle, bone, and adipose tissue), and endoderm (e.g., respiratory or gastrointestinal epithelium). They are most commonly located in the sacrococcygeal region, gonads (ovaries and testes), and midline structures such as the mediastinum and retroperitoneum.

The occurrence of a primary teratoma in the liver is a pathological rarity. The liver is not a typical site for germ cell rests, making the pathogenesis of primary hepatic teratomas a subject of debate. The first case was described by Friedreich in 1898 [1], and since then, only around 28 cases have been reported in the English literature [2,3]. These tumors show a bimodal age distribution, with a majority occurring in infants and children, and only a handful of cases reported in adults [4,5]. The clinical presentation is often non-specific, typically involving abdominal pain or a palpable mass, as in the case we present. Radiological evaluation, particularly computed tomography (CT) and magnetic resonance imaging (MRI), plays a crucial role in diagnosis by revealing the characteristic heterogeneous composition of the tumor. This case report aims to contribute to the sparse literature on this unusual entity by detailing the clinical, radiological, and pathological findings of a primary hepatic teratoma in a 20-year-old female.

## Case presentation

Clinical presentation

A 20-year-old female presented to the outpatient department with a chief complaint of intermittent, dull, aching pain in the upper abdomen for five months. The pain was non-radiating and not associated with posture or food intake. There was no history of fever, jaundice, vomiting, weight loss, or alteration in bowel habits. Her past medical and surgical history was unremarkable.

On general physical examination, the patient was well-built and well-nourished. Vital signs were stable. Abdominal examination revealed a non-tender, firm, palpable mass in the right hypochondrium, which moved slightly with respiration. There was no hepatosplenomegaly or free fluid. Routine hematological and biochemical investigations, including complete blood count, liver function tests (serum bilirubin, alanine aminotransferase, aspartate aminotransferase, alkaline phosphatase), and serum alpha-fetoprotein (AFP) levels, were all within normal limits. Routine laboratory investigations, including complete blood count, liver function tests, and serum tumor markers, were within normal limits (Table 1).

**Table 1:** Laboratory investigations of the patient. AST: aspartate aminotransferase; ALT: alanine aminotransferase; SGOT: serum glutamic oxaloacetic transaminase; SGPT: serum glutamic pyruvic transaminase; β-hCG: β-human chorionic gonadotropin.

Test	Patient value	Reference range	Interpretation
Hemoglobin	12.8 g/dL	12–16 g/dL	Within normal limits
Total leukocyte count	7,200/mm³	4,000–11,000/mm³	Normal
Platelet count	2.5 × 10⁵/mm³	1.5–4.5 × 10⁵/mm³	Normal
Total bilirubin	0.8 mg/dL	0.2–1.2 mg/dL	Normal
AST (SGOT)	28 U/L	5–40 U/L	Normal
ALT (SGPT)	32 U/L	5–45 U/L	Normal
Alkaline phosphatase	112 U/L	44–147 U/L	Normal
Serum albumin	4.2 g/dL	3.5–5.2 g/dL	Normal
Serum alpha-fetoprotein	3.4 ng/mL	<10 ng/mL	Normal
β-hCG	<2 mIU/mL	<5 mIU/mL	Normal
Serum calcium	9.4 mg/dL	8.5–10.5 mg/dL	Normal

Radiological findings

**Figure 1:**
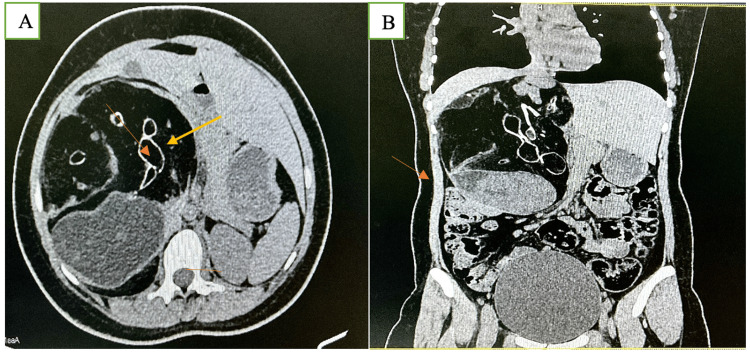
Axial CT images of a primary hepatic teratoma. In the provided CT images of a hepatic mass (presumably a mature teratoma), the arrows in image A mark specific characteristic components. The yellow arrow points toward the fat component of the lesion, seen as hypodense (dark) areas on non-contrast CT, consistent with mature adipose tissue within the teratoma. The orange arrowhead indicates calcification, appearing as a hyperdense (bright white) speck—typical of teeth or bone-like structures often found in teratomas. The brown arrow highlights a fluid or soft tissue density, potentially corresponding to sebaceous or keratinous material, or early solid elements. In image B (coronal post-contrast CT), the orange arrow likely points to an enhancing septation or mural nodule, representing the Rokitansky protuberance, a diagnostic feature comprising solid elements like hair follicles or glandular tissue. These areas enhance with contrast and are key in confirming the diagnosis of a complex teratoma.

Given the palpable abdominal mass, a contrast-enhanced CT (CECT) of the abdomen and pelvis was performed. Non-contrast images revealed a large, well-defined mass lesion measuring approximately 10.2 x 5.3 cm within the liver parenchyma. The mass was notably heterodense, containing areas of macroscopic fat (appearing as low attenuation), multiple irregular calcifications, and soft-tissue components. Post-contrast images showed mild enhancement of the solid components and the cyst walls. The mass exerted a significant mass effect, displacing adjacent intra-abdominal vessels and hepatic parenchyma, but no clear evidence of invasion was noted. The radiological features were highly suggestive of a complex benign lesion, with a mature teratoma being the primary differential diagnosis. Figure 1 shows a large, well-defined multiloculated lesion involving the right lobe of the liver, containing areas of fat and fluid attenuation with multiple internal septations, consistent with a complex cystic mass. In the non-contrast CT image (Figure 1A), the characteristic admixture of fat (hypodense regions), soft tissue, and coarse calcifications (hyperdense foci) can be appreciated, which are typical features of a teratomatous lesion. The post-contrast CT image (Figure 1B) demonstrates enhancing septations and solid nodular components, including a Rokitansky protuberance, further highlighting the heterogeneous and organized internal architecture of the mass. These combined imaging features are suggestive of a primary hepatic teratoma. A few calcific foci were noted within the lesion. Solid nodular lesions were noted along the wall and internal septations.

**Figure 2:**
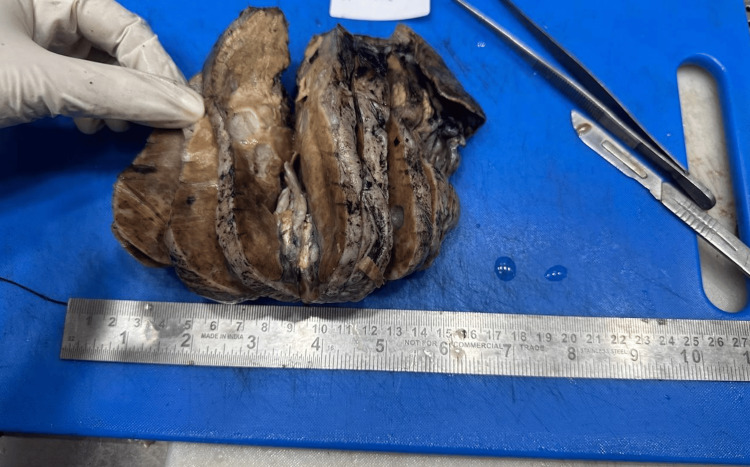
Gross (macroscopic) pathology of the resected hepatic teratoma.

The gross specimen showed a well-circumscribed, multilobulated hepatic mass measuring approximately 10 cm in greatest dimension. On cut section, the lesion demonstrated a heterogeneous appearance with solid and cystic areas containing greasy, sebaceous material, yellowish adipose tissue, and firm calcified components. Focal areas with hair-like material and cartilage-like firmness were noted, consistent with a teratomatous lesion. The surrounding hepatic parenchyma appears compressed but uninvolved, supporting the diagnosis of a primary, well-encapsulated hepatic tumor (Figure 2).

**Figure 3:**
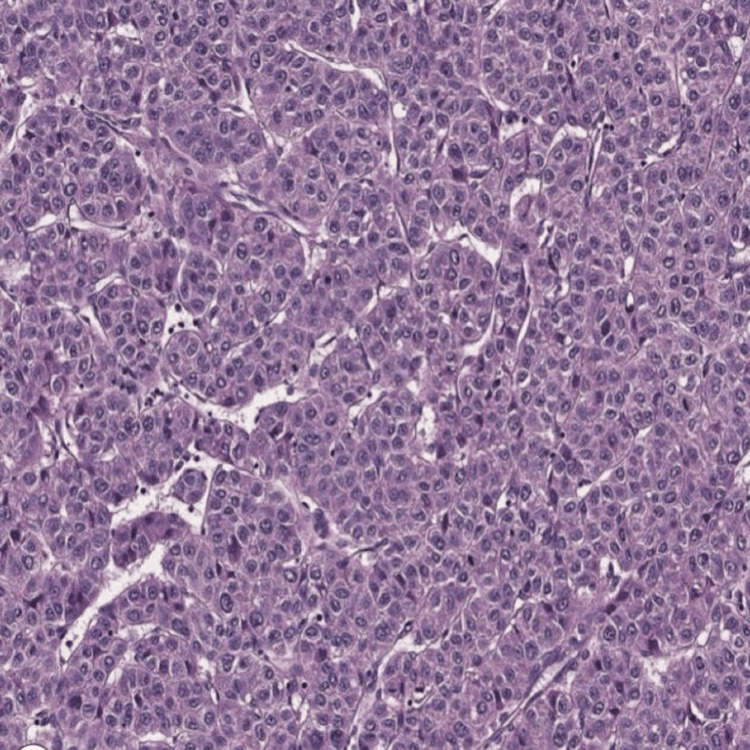
Microscopic (histopathological) features of the hepatic teratoma (hematoxylin & eosin stain).

Microscopic examination revealed a disorganized admixture of well-differentiated tissues derived from multiple germ layers. Ectodermal elements included stratified squamous epithelium with associated skin appendages. Mesodermal components were represented by mature bone, adipose tissue, and fibrous stroma, while endodermal differentiation was evidenced by respiratory-type epithelium. No immature elements, atypia, or malignant transformation were identified. These features confirm the diagnosis of a mature cystic teratoma of the liver and correlate well with the radiological and gross pathological findings (Figure 3).

Management and histopathological outcome

Based on the radiological findings and the patient's symptoms, a decision was made for surgical intervention. The patient underwent an uncomplicated hepatic resection. The gross specimen was a cystic and solid mass containing greasy, sebaceous material, hair, and areas of calcification. Microscopic examination confirmed the diagnosis of a mature teratoma, demonstrating well-differentiated tissues, including stratified squamous epithelium with skin appendages (ectoderm), mature adipose tissue and cartilage (mesoderm), and respiratory-type epithelium (endoderm). There was no evidence of immature or malignant components. The patient had an uneventful postoperative recovery and was discharged in stable condition.

## Discussion

Primary hepatic teratoma is an exceedingly rare entity and often presents a diagnostic challenge due to its nonspecific clinical features. In our case, the patient presented with abdominal discomfort and a palpable mass, accompanied by normal serum tumor markers and characteristic radiological features, consistent with previously reported cases. The pathogenesis of hepatic teratomas is not fully understood. The most widely accepted theory suggests abnormal migration of primordial germ cells during embryogenesis, wherein pluripotent cells destined for the gonads are inadvertently arrested within the developing liver and later give rise to teratomatous tissue [6]. Another embryologic explanation proposes the misplacement or persistence of ectopic pluripotent cells during the early development of the foregut, which is closely involved in hepatic formation [7,8].

Although hepatic teratomas are predominantly reported in infants and young children, a smaller proportion of cases occur in adults. In the available literature, adult cases remain distinctly uncommon, yet a pattern of occurrence in young female adults is noted. For example, Jaklitsch et al. reported a mature cystic teratoma in an adult patient [1], Kovalenko et al. described a similar presentation in a young woman [3], and Nguyen et al. documented an immature teratoma in an adult female [4]. These findings align closely with our case. By contrast, adult male cases are far more unusual, with scattered isolated reports such as those described by Malek-Hosseini et al. and Ramkumar et al. [9,10].

Across nearly all reported cases, the clinical presentation reflects mass effect, typically manifesting as abdominal pain or distension. Imaging plays a crucial role in diagnosis. CT is particularly useful for identifying the characteristic combination of fat, calcification, and soft tissue components, which collectively serve as a strong radiologic indicator of teratoma [1,3,4]. Importantly, normal AFP and β-human chorionic gonadotropin (β-hCG) levels, as seen in our patient and widely reported in mature teratomas, help differentiate these lesions from malignant germ cell tumors or primary hepatic malignancies [1,3,4].

Complete surgical excision remains the standard treatment and is considered curative for mature teratomas [1-4,9,10]. Chemotherapy or radiotherapy is not required unless the lesion demonstrates immature histology or malignant transformation, which is exceedingly rare [9]. Long-term prognosis following full resection is excellent, and recurrence is uncommon. Consistent with findings in previously reported adult cases, our patient underwent successful surgical resection and is anticipated to have a favorable outcome [11,12].

Literature summary

Table 2 summarizes key characteristics of previously reported cases of primary hepatic teratoma, highlighting the rarity of this condition.

**Table 2:** Summary of reported cases of primary hepatic teratoma in the literature.

Study/author(s)	Patient age/sex	Presentation	Key imaging findings	Treatment	Histology
Jaklitsch et al. (2019) [1]	Adult (not specified)	Abdominal mass, discomfort	CT: Complex cystic-solid mass with fat + calcification	Surgical resection	Mature teratoma
Ravikumar et al. (2018) [2]	Newborn/F	Abdominal distension at birth	CT: Mass in hepatoduodenal ligament; vascular/biliary involvement	Complex surgery with reconstruction	Mature teratoma
Kovalenko et al. (2021) [3]	Adult/F	Right upper quadrant pain	CT/MRI: Multiloculated hepatic mass with fat-containing areas	Surgical excision	Mature teratoma
Nguyen et al. (2023) [4]	Adult/F	Epigastric pain & weight loss	CT/MRI: Mixed cystic-solid lesion; limited fat components	Surgical resection + pathology review	Immature teratoma
Malek-Hosseini et al. (2010) [9]	Adult/M	Abdominal swelling	CT: Large complex mass with cystic and solid components	Surgical resection	Immature teratoma
Ramkumar et al. (2018) [10]	Adult/M	Acute abdomen due to rupture	CT: Ruptured complex hepatic mass with fat and calcification	Emergency surgical resection	Mature teratoma
Current case (2024)	20 years/F	Intermittent abdominal pain	CT: Well-defined multiloculated mass with fat, fluid, calcification, and internal septations	Curative surgical excision	Mature teratoma

The presentation and management of this case carry several important clinical implications. First, primary hepatic teratoma should be considered in the differential diagnosis of a complex hepatic mass, even in adults, particularly when imaging demonstrates the characteristic triad of fat, calcification, and soft-tissue components. Early recognition of these features can support a presumptive diagnosis without the need for invasive biopsy. Second, identifying the likely benign nature of a mature teratoma and correlating it with normal tumor markers assists in preoperative planning and guides surgeons toward curative resection rather than potentially hazardous or unnecessary interventions. Third, the involvement of a multidisciplinary team, including hepatobiliary surgeons, radiologists, and pathologists, is crucial for accurate diagnosis and optimal management. Finally, effective patient counseling is essential, as understanding the typically benign and treatable nature of the condition can alleviate anxiety and support shared decision-making regarding surgical treatment.

## Conclusions

Primary hepatic teratoma is a rare but important diagnostic consideration when evaluating a complex hepatic mass in a young individual. A high index of suspicion, coupled with characteristic radiological identification of fat and calcification within the lesion, can lead to a strong preoperative diagnosis. The absence of elevated tumor markers further supports the diagnosis of a benign mature teratoma. As demonstrated by this case and its consistency with previous reports, complete surgical resection is the treatment of choice and provides definitive diagnosis and cure. This case adds to the limited body of knowledge on this unusual entity and reinforces the critical role of multimodal imaging and a multidisciplinary approach in its diagnosis and management.
